# Effects of KEAP1 Silencing on the Regulation of NRF2 Activity in Neuroendocrine Lung Tumors

**DOI:** 10.3390/ijms20102531

**Published:** 2019-05-23

**Authors:** Angelo Sparaneo, Federico Pio Fabrizio, Annamaria la Torre, Paolo Graziano, Massimo Di Maio, Andrea Fontana, Michele Bisceglia, Antonio Rossi, Stefano Pizzolitto, Giovanna De Maglio, Antonio Tancredi, Franco Grimaldi, Teresa Balsamo, Flavia Centra, Maria Carmina Manzorra, Domenico Trombetta, Angela Pantalone, Antonio Bonfitto, Evaristo Maiello, Vito Michele Fazio, Lucia Anna Muscarella

**Affiliations:** 1 Laboratory of Oncology, Fondazione IRCCS Casa Sollievo della Sofferenza, 71013 San Giovanni Rotondo (FG), Italy; a.sparaneo@operapadrepio.it (A.S.); fp.fabrizio@operapadrepio.it (F.P.F.); a.latorre@operapadrepio.it (A.l.T.); t.balsamo@operapadrepio.it (T.B.); f.centra@operapadrepio.it (F.C.); mc.manzorra@gmail.com (M.C.M.); d.trombetta@operapadrepio.it (D.T.); 2 Unit of Pathology, Fondazione IRCCS Casa Sollievo della Sofferenza, 71013 San Giovanni Rotondo (FG), Italy; p.graziano@operapadrepio.it (P.G.); a.bonfitto@operapadrepio.it (A.B.); 3 Department of Oncology, University of Turin, Ordine Mauriziano Hospital, 10128 Turin, Italy; massimo.dimaio@unito.it (M.D.M.); 4 Unit of Biostatistics, Fondazione Casa Sollievo della Sofferenza Hospital, 71013 San Giovanni Rotondo (FG), Italy; a.fontana@operapadrepio.it (A.F.); 5 Anatomic Pathology, School of Biomedical Sciences, Etromapmax Pole, 71010 Lesina (FG), Italy; bismich@alice.it (M.B.); 6 Department of Onco-Haematology, Fondazione IRCCS Casa Sollievo della Sofferenza, 71013 San Giovanni Rotondo (FG), Italy; arossi_it@yahoo.it (A.R.); e.maiello@libero.it (E.M.); 7 Department of Pathology, Azienda Sanitaria Universitaria Integrata di Udine, 33100 Udine, Italy; stefano.pizzolitto@asuiud.sanita.fvg.it (S.P.); demaglio.giovanna@aoud.sanita.fvg.it (G.D.M.); 8 Thoracic Surgery Unit, Fondazione IRCCS “Casa Sollievo della Sofferenza”, 71013 San Giovanni Rotondo (FG), Italy; antoniotancredi@virgilio.it (A.T.); 9 Department of Endocrinology, Azienda Sanitaria Universitaria Integrata di Udine, 33100 Udine, Italy; doctor@francogrimaldi.it (F.G.); 10 Genetic and Clinic Pathology Unit, University Campus Bio-Medico of Rome, 00128 Rome, Italy; angela.pantalone@libero.it (A.P.); FAZIO@unicampus.it (V.M.F.)

**Keywords:** Lung Carcinoid, KEAP1, NRF2, methylation, mutation, outcome

## Abstract

Background. The KEAP1/NRF2 pathway has been widely investigated in tumors since it was implicated in cancer cells survival and therapies resistance. In lung tumors the deregulation of this pathway is mainly related to point mutations of *KEAP1* and *NFE2L2* genes and *KEAP1* promoter hypermethylation, but these two genes have been rarely investigated in low/intermediate grade neuroendocrine tumors of the lung. Methods. The effects of *KEAP1* silencing on NRF2 activity was investigated in H720 and H727 carcinoid cell lines and results were compared with those obtained by molecular profiling of KEAP1 and *NFE2L2* in a collection of 47 lung carcinoids. The correlation between methylation and transcript levels was assessed by 5-aza-dC treatment. Results. We demonstrated that in carcinoid cell lines, the *KEAP1* silencing induces an upregulation of NRF2 and some of its targets and that there is a direct correlation between *KEAP1* methylation and its mRNA levels. A *KEAP1* hypermethylation and Loss of Heterozygosity at *KEAP1* gene locus was also observed in nearly half of lung carcinoids. Conclusions. This is the first study that has described the effects of *KEAP1* silencing on the regulation of NRF2 activity in lung carcinoids cells. The epigenetic deregulation of the KEAP1/NRF2 by a KEAP1 promoter hypermethylation system appears to be a frequent event in lung carcinoids.

## 1. Background

The KEAP1 (Kelch-like ECH-associated protein 1)/NRF2 (Nuclear factor (erythroid-derived 2)-like 2) is a master pathway regulator of antioxidants and cellular stress responses and is clearly implicated in neoplastic progression and resistance of tumor cells against chemo- and radio-treatments [1], so the consequent enhancement of NRF2 expression is becoming a new potential target of anticancer therapeutic approaches [2].

In solid tumors, both genetic and epigenetic mechanisms are described to play a significant role in the impairment of KEAP1/NRF2 activity. In lung cancer, promoter *KEAP1* methylation and point mutations in functional domains of *KEAP1* and *NFE2L2* genes are firstly reported in NSCLC [3,4] and then widely described as a specific signature of aggressiveness in many solid tumors, being correlated with overall survival and response of patients to standard treatments [5,6]. On the other side, molecular data regarding the deregulation of redox axis in pulmonary neuroendocrine tumors (lung NETs) are limited, even if several important indications have emerged in recent years. Specifically, the *KEAP1* genetic alterations were described by Fernandez-Cuesta et al. as a new uncovered molecular hallmark of the Lung LCNEC (Large Cell Neuroendocrine Carcinoma) group with adenocarcinoma-like features [7]. A lack of evidence suggest an important question that needs to be addressed when considering the alterations of KEAP1-NRF2 pathway in the other lung NETs and the impact of NRF2-related targets in the epigenetic context of lung neuroendocrine carcinoma.

Carcinoids are rare neuroendocrine tumors of the lung with a low mutation rate and few recurrently mutated genes, mainly clustered in the genes involved in chromatin remodeling events, such as acetylation and methylation [8,9]. Epigenetic marks in carcinoids are poorly investigated and DNA methylation of few genes are reported to have a prognostic value in the context of typical and atypical lung carcinoids [10,11].

No somatic mutations in *KEAP1* and *NFE2L2* genes were identified by a unique WES study performed to date in a limited cohort of patients [8], and no investigations have been performed until now about the epigenetic status of the *KEAP1* promoter region both in typical (TC) and in atypical carcinoids (AC), [12].

We aim to investigate the effects of KEAP1 on NRF2 modulations and the existing molecular background of this pathway in low/intermediate grade lung NETs by evaluating the effects of *KEAP1* silencing on NRF2 and its target activity in carcinoid cell lines. Additionally, we perform the first epigenetic profile of *KEAP1* promoter region in lung carcinoid tissues together with the genetic screening of the *KEAP1* and *NFE2L2* genes and LOH analysis.

## 2. Results

### 2.1. The KEAP1 Silencing Affects the NRF2 and Expression Levels of TXNRD1, AKR1C1 and NQO1 in Carcinoid Lines

**Figure 1:**
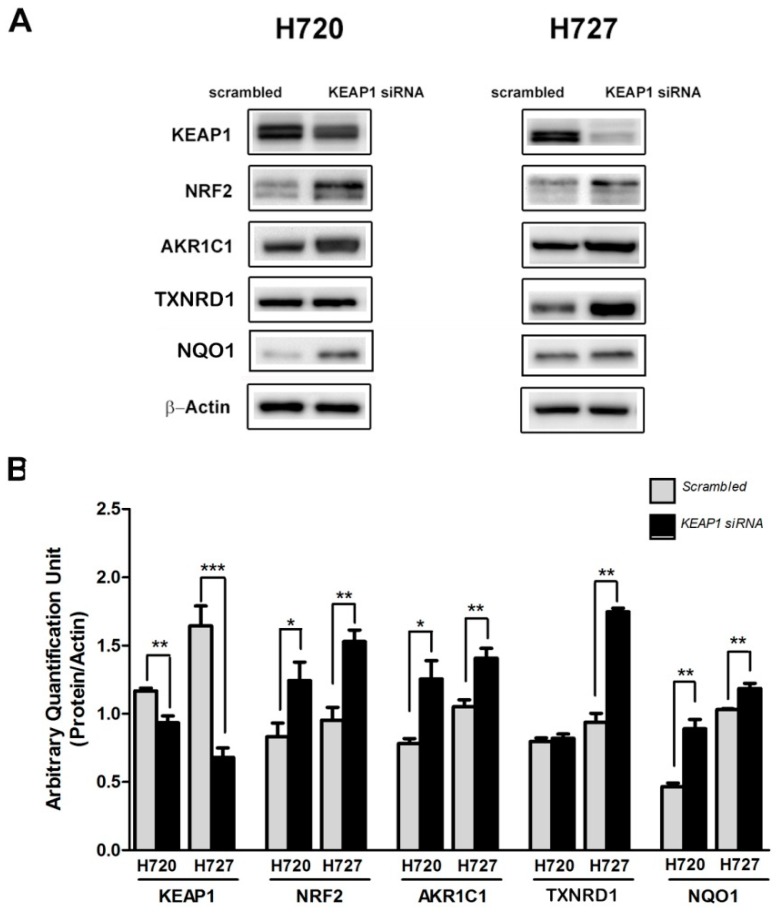
(**A**) Representative Western Blots analysis showing expression levels of KEAP1, NRF2, AKR1C1 and TXNRD1 in H720 and 727 cell lines after *KEAP1* inhibition by specific *KEAP1* siRNA. Scrambled siRNA was performed as the control. (**B**) Histograms show the expression levels of the proteins normalized to actin (*N* = 4). No overexposure was done. The grouping of gels/blots were cropped from different parts of the same gels. * *p* < 0.05, ** *p* < 0.001, *** *p* < 0.0001 *t*-test.

NRF2 activation was determined in H720 cells with transient silencing of the *KEAP1* gene. Following KEAP1 siRNA transfection, the protein level of NRF2 showed a significant up-regulation compared to NRF2 levels of Scrambled siRNA transfection, both in H720 and in H727 cell lines (* *p* < 0.05 and *p* < 0.001, *t*-test). Consistent with the increase of NRF2 levels in both cell lines, in the H720 cell line AKR1C1 and NQO1 ARE-dependent genes were significantly higher in KEAP1-Knock Down cells than SCR cells, whereas in the H727 cell line we observed a significant increase of TXNRD1, AKR1C1 and NQO1 protein levels after *KEAP1* silencing (Figure 1).

### 2.2. Restoration of KEAP1 Expression Correlates with KEAP1 P1 Region Demethylation by 5-aza-dC Treatment in Carcinoid Cell Lines

**Figure 2:**
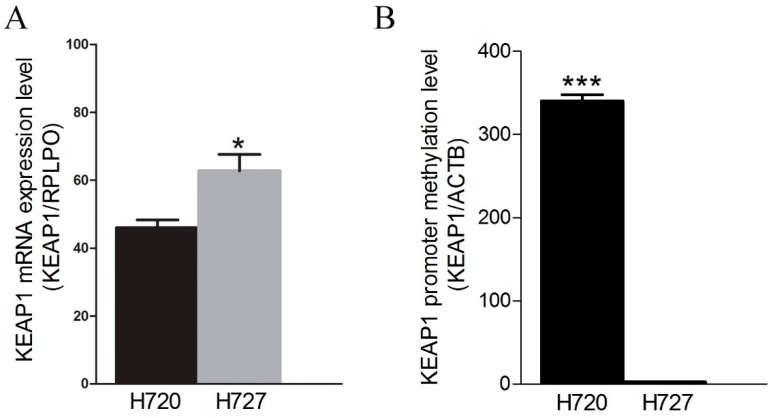
(**A**) Expression level analysis (± standard error mean) of the *KEAP1* gene in H720 and H727 determined by RT-qPCR. The relative quantification was expressed as the ratio marker (KEAP1/RPLPO). (**B**) Methylation levels (± standard error mean) of *KEAP1* in H720 and H727 determined by qMSP (*N* = 4). * *p* < 0.05, *** *p* < 0.0001 *t*-test.

A genetic and epigenetic H720 and H727 was assessed and no mutations in *KEAP1* or *NFE2L2* genes were found. By contrast, an aberrant *KEAP1* promoter methylation was observed in the H720 cell line compared with H727, which reflects the KEAP1 mRNA expression level in the two lines (Figure 2A).

**Figure 3:**
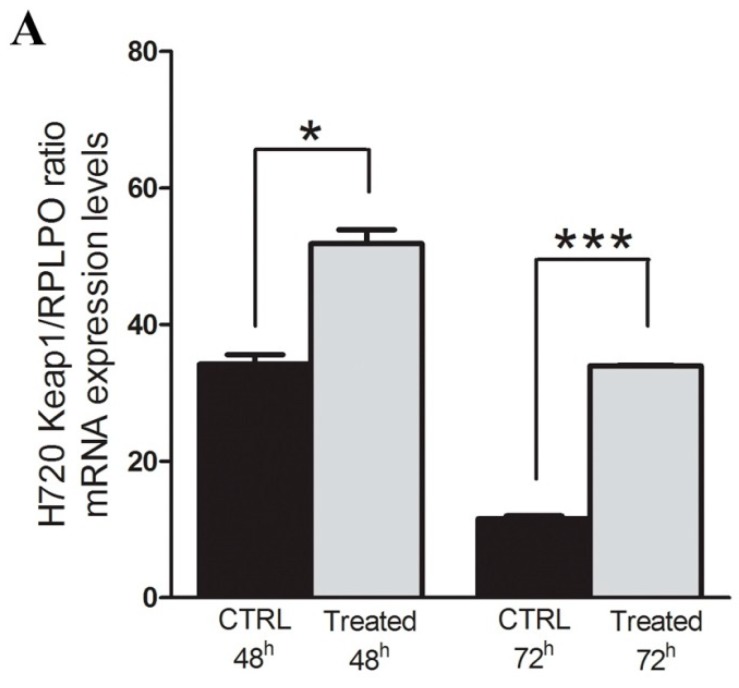

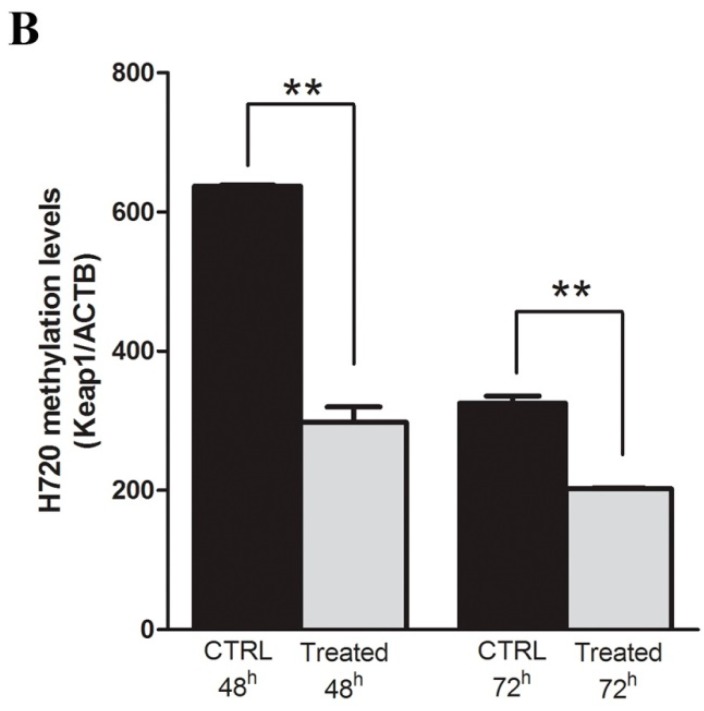
(**A**) Changes in KEAP1 mRNA transcript levels in the H720 cell line before (Untreated) and after treatment with 5-aza-dC at 24 h and 48h. Error bars indicate the standard deviation of three different experiments. * *p* < 0.01, *t*-test. (**B**) Changes in *KEAP1* promoter methylation levels in the H720 cell line by quantitative methylation real-time PCR before (CTRL) and after treatment with 5-aza-dC at 48 and 72 h. Error bars indicate the standard deviation of three different experiments. * *p* < 0.05, *t*-test.

The real effect of promoter CpG methylation on the modulation *KEAP1* mRNA expression level was observed in the atypical carcinoid H720 hypermethylated cell line under 5-aza-dC treatment. The variation of methylation and KEAP1 mRNA level was examined before and during the demethylating treatment (Figure 3A) by RT-qPCR and qMSP. A progressive significant increase of the KEAP1 transcript abundance was observed after 24h (*p* < 0.01) and 48h (*p* < 0.01) concomitantly with a decreased *KEAP1* promoter methylation at both time points (*p* < 0.01), (Figure 3B).

### 2.3. KEAP1 Promoter Region Hypermethylation are Frequent Epigenetic in Lung Carcinoids, Whereas Point Mutations are Absent

The *KEAP1* promoter methylation level was evaluated on DNA obtained from a total of all lung carcinoid tissues and 12 normal lung tissues from non-neoplastic patients (NL). The median values and Inter Quartile Ranges (IQR) for *KEAP1*/*ACTB* ratios were 0.0 (0-0) for NL, as a consequence all positive values in tumor tissues were considered to be equal to a hypermethylated status. In TC *KEAP1* methylation was detected in 15 out of 30 cases (50%), while in AC methylation was detected in 8 out of 17 cases (47%). No statistically significant differences in methylation levels assessed by qMSP were detected between the TC and AT groups.

To ascertain the status of the *KEAP1* locus we genotyped 17 tumors (5 ACs and 12 TCs) of the cohort and their corresponding peripheral blood/normal tissues using the D19S865, DM1, D19S906 and D19S2840 microsatellite markers located at the 19p13.2 locus. As a result, 3/5 AC (60%) and 6/12 (50%) TC groups demonstrated LOH for at least one of the markers (Table 1). An alternative mechanism to achieve the deregulation of *KEAP1* would be the occurrence of somatic mutations in our Carcinoid samples. Despite the literature evidence, to exclude this possibility we analyzed all carcinoid cohorts, searching for somatic alterations in the whole coding region of the *KEAP1* gene and in the Neh2 domain of the *NFE2L2* gene. This analysis did not reveal any sequence variations that would likely result in a functional alteration in the KEAP1 or NRF2 proteins.

**Figure 4:**
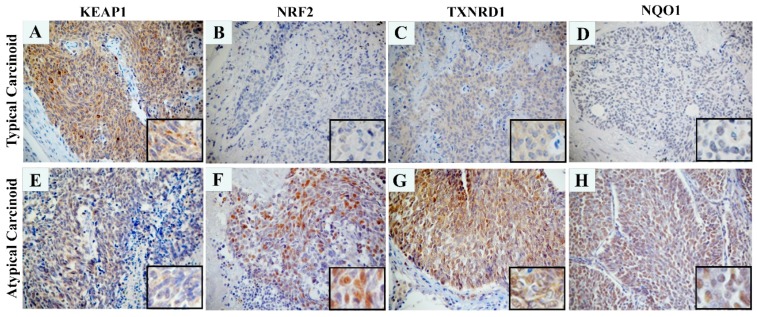
Immunohistochemistry analysis showing KEAP1, NRF2, TXNRD1 and NQO1 proteins expression in one typical (unmethylated, **A**–**D**) and one atypical (methylated, **E**–**H**) lung tissue from two different carcinoid affected patients. 20× and 40× magnification. A strong KEAP1 cytoplasmatic staining was observed in a typical unmethylated lung carcinoid tissue with a mild nuclear NRF2 and cytoplasmatic TXNRD1 and NQO1 staining, whereas an inverse protein staining pattern was observed in an atypical methylated lung carcinoid tissue.

To assess the possible correlation between the KEAP1 protein levels in carcinoid and the epigenetic silencing of the *KEAP1* gene, 11 tumor cases (3 AC and 8 TC) showing *KEAP1* promoter methylation and 3TC tumors without aberrant methylation were analyzed by immunohistochemical analysis. All the areas of the tissue section were evaluated for KEAP1 protein expression. A variable KEAP1 immunoreactivity was observed in the cytoplasm of tumor cells but no statistically significant differences were observed. Only in a few cases was it possible to notice a direct relationship among KEAP1 downregulation and overexpression of NRF2 and its target TXNRD1 and NQO1 (Figure 4).

Patients’ clinical-pathological features analyzed into the study are summarized in Table 2. Overall, the series included 47 patients grouped in 30 TC (64%) and 17 AC (36%). The average patient’s age at the time of diagnosis was 63 years with a range from 29 to 76 years. More than half of the patients were women (55%). All patients underwent curative surgery and all malignant lesions were subjected to staging according to the TNM system (2009 classification). The lymph node involvement at the diagnosis was found only in 5 cases (N1 and N2 patients, 11%). During follow-up, no patients showed a relapse with distant metastasis. After a median follow-up of 92 months, 6 patients (13%) died (1 with CT and 5 with AC), whereas 41 (87%) patients were still alive at the last visit.

The epigenetic status of *KEAP1* was correlated to clinical-pathological features of both typical and atypical lung carcinoids. No significant correlation was found (Table 3 and Table 4). Time-to-event analysis was based on 8 events for disease-free survival and 6 events for overall survival.

Univariate time-to-event analysis showed no significant association between the epigenetic status of *KEAP1* and risk of progression (HR = 3.122; 95% CI 0.630–15.474, *p* = 0.163) and no significant association between the epigenetic status of *KEAP1* and risk of death (HR = 1.769, 95% CI 0.316–9.917, *p* = 0.517), (Supplementary Figure S1). Similar results were obtained in the analysis stratified by histological subtype (Typical vs. Atypical).

## 3. Discussion

Lung Carcinoids belong to the heterogeneous group of malignancies characterized by an indolent rate of growth and progression with a genetic pattern that contains lesions in the classical oncogenes or tumor suppressor genes identified in NSCLCs [13]. By contrast, carcinoids show frequent mutations in chromatin remodeling genes, although molecular alterations are rare in general, suggesting the presence of alternative pathogenic drivers. Epigenetic landscape and promoter methylation events are poorly investigated in lung carcinoids and only few genes were described to date to be involved in this specific context [12]. *RASSF1* promoter hypermethylation was found as the most frequent epigenetic event reported in both typical and in atypical lung carcinoids and it was associated with tumor grade but without a linear correlation between levels of methylation and RASSF1A mRNA or protein content [14]. *MCAM* methylation levels were found to be significantly higher in lung typical carcinoids tumors and were proposed as a useful new molecular biomarker in this tumor type [15]. Finally, in a small series of 5 low-grade lung NETs, aberrant methylation at the 5′-region of the *p15INK4b* gene was observed in 15% of tumors, but not in normal bronchial tissues [16].

To counteract oxidative stress cells, we developed efficient mechanisms of defense. One of the most important mechanisms involves the activation of the Keap1/Nrf2/ARE pathway. NRF2 is a transcription factor that coordinates the transiently cellular redox changes and modulates the cellular defense against toxic and oxidative damage, mitochondrial physiology, differentiation and stem cell maintenance [17]. Upon exposure to stress, NRF2 translocates into the nucleus, where it binds to the antioxidant response element (ARE) located in the promoter regions of many cytoprotective genes such as drug-metabolizing enzymes and antioxidant systems which require NADPH as a cofactor, including aldo-ketoreductase family 1 member C1 (AKR1C1), NAD(P)H:quinone oxidoreductase-1 (NQO1) and thioredoxin reductase (TXNRD1) [18,19]. The deregulation of this pathway along with an increased NRF2 activity was firstly described in NSCLC cells, but was quickly confirmed in many solid tumors. A lot of molecular mechanisms were described to be linked to KEAP1/NRF2 deregulation, such as somatic mutations in *NFE2L2*, *KEAP1* or *CUL3* genes; promoter methylation of *KEAP1*; microRNA-mediated regulation of KEAP1 and NRF2 [2]. Frequent LOH was observed in at 19p13.2, thus confirming the role of *KEAP1* as a tumor suppressor gene [3,6]. Despite of the widely investigations of this pathway in NSCLC and the observed link between many NRF2 target proteins and lung neuroendocrine tumors (lung carcinoid and SCLC), few studies were performed to investigate the link between lung carcinoid and oxidative stress (Table 5), [20].

Here we describe the results obtained from the first comprehensive genetic and epigenetic profile of *KEAP1* and *NFE2L2* genes in a collection of lung carcinoid tumors. No mutations were identified in our cohort, so we can clearly exclude a driver contribution by these type of molecular lesions in this lung NETs. Data produced were in fact similar to those reported by the unique WES study performed to date by Simbolo et al. [9] in a total of 44 cases (23 TC and 14AC samples), and by three previously large published studies [59]. By contrast, the *KEAP1* genetic alterations appear to be strongly related to a high-grade lung NET. Specifically, Fernandez-Cuesta et al. described *KEAP1* point mutations as a specific molecular event associated with pulmonary LCNECs with adenocarcinoma-like features [7]. This last finding was confirmed by a different group which reported a prevalence of *KEAP1-NFE2L2* (31%) alterations in tumors with high neuroendocrine gene expression, mainly co-occurring with *STK11* and *KRAS* genes to exert a synergic role of tumorigenesis enhancement and cancer progression [60].

We decided to investigate the effect of *KEAP1* silencing in lung carcinoids by performing functional investigation in H720 and H727 cell lines. The *KEAP1* silencing studies by siRNAs performed both in H720 and in H727 cell lines strongly indicate that KEAP1 is also a master regulator of NRF2 in lung carcinoids cells. Since NRF2 coordinates the regulation of detoxification and intracellular redox balance in response to oxidative stress, some of the NRF2 targets such as TXNRD1, AKR1C1, and NQO1 have been functionally chosen which are well-known implicated in drug and/or radiation resistance contexts of solid tumors [61]. NQO1 has attracted much attention as a potential target for the treatment of cancer because it has been shown to be frequently expressed at much higher levels in tumors, mainly in lung tumors, relative to adjacent non-neoplastic tissue regions. In addition, NQO1 activity appears to be increased during tumor progression. A clear modulation by KEAP1 on these NRF2-target proteins has been observed and confirms the control activity by KEAP1 protein to the detoxifying pathway NRF2-related in carcinoids cells.

A direct correlation between *KEAP1* epigenetic silencing and transcript level was also demonstrated in the *KEAP1* hypermethylated H720 cell line under the demethylating treatment with 5-aza-2-deoxycytidine. As previously reported in other tumors, a clear recovery of the KEAP1 transcription level was in fact observed and confirmed the inverse correlation between KEAP1 expression and CpGs island hypermethylation [2]. The epigenetic silencing by *KEAP1* hypermethylation in lung carcinoids was then investigated in carcinoid tissues from 47 patients. As results, aberrant *KEAP1* methylation of the gene P1 promoter region [4,13] was identified in both TC and AC samples at a high frequency of about 50% without any significant difference in methylation levels detected by comparing TCs with ACs. LOH at the gene locus was also demonstrated in 50–60% of the tumors. Overall 24% of the tumors showed two alterations (LOH and promoter methylation) in *KEAP1*, suggesting that both copies of the gene might be inactivated.

The concordance between methylation levels and protein expression was also investigated by immunohistochemical analysis in 11 tumor cases showing *KEAP1* promoter methylation (3 AC and 8 TC) and 3 TCs without methylation. A variable KEAP1 and NRF2 immunoreactivity were observed in the cytoplasm of tumor cells, but no statistically significant difference was appreciated. Only in a few cases was it possible to notice a direct relationship between *KEAP1* down-regulation and overexpression of NRF2 and its targets NQO1 and TXNRD1 but data were not adequate to provide any conclusions. The lack of correlation between KEAP1 methylation and protein levels should be mainly explained taking into account that 1) other transcriptional or post-transcriptional events may concur in *KEAP1* regulation; 2) It is plausible that the use of a non-specific qMSP assay does not allow us to discriminate among really functional and non-functional CpGs of the P1 promoter island, since it covers simultaneously all the CpGs of *KEAP1* P1 promoter region, so it should be difficult to appreciate the effective functional level of methylation to correlate with protein expression. From a translational point of view, these considerations should also justify the lack of a clear correlation between methylation and disease course, both in TC and AC. Currently, there is no consensus on the best technique for *KEAP1* methylation assessment. How many and which CpG sites of *KEAP1* promoter should be analyzed remains a controversial issue in a translational context [2]. However, a more strong impact on KEAP1 expression level should be hypothesized for the CpG sites located in the critical sub-region closer to the transcription start site (TSS) of *KEAP1* gene which contains putative binding sites for Sp1 and AP2 transcription factors [22,52,53].

Despite all these considerations, the *KEAP1* silencing studies support the hypothesis that KEAP1 acts in lung carcinoids by modulating the levels of NRF2 and the NRF2-detoxification enzymes TXNRD1 and NQO1.

Looking at a possible correlation between methylation and clinic-pathological parameters, it was observed that the degree of promoter methylation showed a trend of association with a higher risk of progression, which is difficult to confirm due to the indolent course of disease of lung carcinoid and a large number of cases required for a validation in an independent cohort. Anyhow, the general observation of a deregulation of KEAP1/NRF2 axis in lung carcinoid provided the opportunity to consider new therapeutic approaches that directly involve epi-drugs or drugs affecting redox processes to support the chemo and radio-therapeutic options strictly related to the generation of ROS [62]. More interestingly, it should be noticed that the NRF2 is a master transcription factor that regulates a wide variety of cellular proteins and recently it has been reported to indirectly up-regulate mTOR activity by increasing the intermediary protein RAGD, an activator of mTOR and positively modulate the transcription of mTOR in cells with abnormally active PI3K signaling [63,64]. The link between the NRF2 system of sensing environmental stress and mTOR may suggest a possible role of KEAP1/NRF2 deregulation observed in lung carcinoids in prediction to response to mTOR pharmacological inhibition in these tumors [65]. On the other hand, the high prevalence of the PI3K pathway deregulation in cancer cells could offer the opportunity to selectively modulate this pathway via NRF2 in non-tumor cells, an approach that deserves exploration [5,66]. Finally, it must be take into account that NRF2 levels can be regulated in a KEAP1-indipendent way by carcinoid tumor-related oncogenes, so their profile should impact and must be monitored to predict a tumor response to pharmacological treatments [67].

Actually, some chemicals were also reported as NRF2 modulators, although the molecular mechanisms through which these compounds inhibit NRF2 were not been fully elucidated. Since NRF2 inhibitors are well-known as suppressors of the proliferation of cancer cells, many questions related to the NRF2 inhibitors remain unsolved about the real utility in clinical trials, especially in the context of lung cancer. Many NRF2 modulators were discovered by starting from natural compounds, such as brusatol [68] which contributes to the decrease of NRF2 expression at both transcript and protein levels in the abovementioned cells, luteolin (3′,4′,5,7-tetrahydroxyflavone), [69] and all trans-retinoic acid (ATRA), an active metabolite of vitamin A, which represses NRF2 activity by inhibiting its capability to bind to ARE sequences of its target genes [70]. In addition, semisynthetic and synthetic compounds were investigated in clinical trials, such as ML385, a thiazole-indoline compound which targets the DNA-binding region of NRF2 protein (Neh1 domain) and successively inhibits the activation of its downstream target genes. In preclinical models of *KEAP1* mutated lung cancer cells, it was observed that ML385 plus carboplatin enhances the efficacy of anti-tumor activity of chemotherapic compounds [71]. Clobetasol propionate was identified as the most potent Nrf2 inhibitor with strong in vivo and in vitro effects on the suppression of the anchorage-independent growth of lung tumors harboring *KEAP1* mutations, alone or in combination with rapamycin [72].

In general, there are no currently specific inhibitors of NRF2 available in clinical practice of lung cancer patients to pharmacologically target NRF2 pathway because of all of them show poor results for anticancer therapy and additional studies are needed to gain insights into the mechanism of Nrf2 modulation.

## 4. Methods

### 4.1. Cell Lines

The H720 atypical carcinoid cell line and the H727 typical carcinoid cell line were purchased from American Type Culture Collection (ATCC, Manassas, VA, USA) and ECACC (Porton Down, Salisbury, UK), respectively. Cells were cultured in RPMI 1640 medium, supplemented with 10% FBS.

### 4.2. KEAP1 Silencing by siRNA

KEAP1 siRNA duplexes specific for human gene *KEAP1* (NM_012289) were purchased from Thermo Scientific (Carlsbad, CA, USA), (Silencer Select, s18981). A scrambled siRNA was used as a negative control (CTRL siRNA), (Silencer Select, Negative Control) and purchased from Thermo Scientific. RNA interference (RNAi) experiments in carcinoid cells were performed by transient transfection for 48h. The RNAiMAX Lipofectamine (Thermo Fisher, Invitrogen) transfection protocol was used. Cells were analyzed for KEAP1 expression by Western blot analysis after 48 h.

### 4.3. Western Blot Analysis

Cells were lysed with RIPA buffer and proteins were separated on 13% SDS-PAGE and transferred onto a PVDF membrane. The membrane was stained immunochemically using anti-KEAP1, NRF2, NQO1, TXNRD1 (Proteintech, Chicago, IL, USA), and by using anti-AKR1C1 (Abnova, Taiwan). H720 and H727 cells were cultured in RPMI 1640, supplemented with 10% FBS and antibiotics. Equal amounts of protein lysates (35 μg) were resolved by SDS-PAGE (13% acrylamide) under reducing conditions and transferred onto PVDF membranes (Immobilon PVDF, Millipore, Billerica, MA, USA). After transfer, PVDF membranes were blocked with 5% non-fat milk and incubated with primary antibody overnight at 4 °C. Rabbit anti-NRF2 (Proteintech), rabbit anti-NQO1 (Proteintech), rabbit anti-TXNRD1 (Proteintech), mouse anti-AKR1C1 (Abnova) and anti-Actin (Sigma-Aldrich, St. Louis, MO, USA) were incubated overnight at 4 °C. PVDF membranes were incubated with ECL plus solution following the manufacturer’s instructions (Thermo Fisher Scientific, Rockford, IL, USA). Chemiluminescence signals were acquired by ChemiDoc XRS (Biorad, Hercules, CA, USA).

### 4.4. In Vitro 5-Aza-2′-deoxycytidine (5-aza-dC) Treatment

Treatment with the 5-aza-2′-deoxycytidine (DAC), was performed using a concentration of 5µM (Sigma-Aldrich) with fresh medium for 24 h and 48 h. Cells were harvested at both time points for DNA and RNA isolation to perform *KEAP1* methylation and gene expression analysis.

### 4.5. RNA Extraction, Reverse Transcription and Quantitative Real-Time PCR (qRT-PCR)

RNA quality was measured by using 2100 Expert Analyzer (Agilent Technologies, Santa Clara, CA, USA) and RNA with RIN (RNA Integrity Number) ≥7.0 was processed. RNA concentrations were quantified by the Nanodrop spectrophotometer. cDNA was synthesized from 1 μg of total RNA using SuperScript III (Thermo Fisher, Invitrogen division) and qRT-PCR was by TaqMan^®^ Universal PCR Master Mix (Thermo Fisher, Life Technologies division), and TaqMan™ Gene Expression Assay with specific TaqMan probe assay (listed in Supplemental Table S1) on ABI PRISM 7900 Sequence detection system (Thermo Fisher, Applied Biosystems). Serial dilutions (1 × 10^6^–1 × 10^2^ copies) of plasmid product were used to build a standard curve and mRNA levels in each sample were determined using a relative quantification method as the ratio of the *KEAP1* expression level to the *RPLPO* expression.

### 4.6. Patients and Tissue Samples

The patients’ clinical and pathological data including pathological TNM staging, site of the lesion, grading, age, gender, and follow-up data were collected and are shown in Table 2.

We analyzed lung carcinoid tissues obtained as fresh frozen specimens/FFPE tissues from a total of 47 patients (30 Typical Carcinoids and 17 Atypical Carcinoids) surgically treated at IRCCS Casa Sollievo della Sofferenza, San Giovanni Rotondo (FG), Italy and Santa Maria della Misericordia”, Udine, Italy. As control, 12 histologically confirmed normal lung tissues distant from tumor were analyzed.

All tissue specimens from patients were fixed in neutral-buffered formalin prior to paraffin embedding (FFPE samples). Sections, 3-μm-thick, were cut from FFPE tissue blocks and marked by a pathologist on H&E-stained slides to verify tumor cellularity. DNA was extracted from corresponding unstained 12-µm-thick section by manual microdissection and subsequently isolated by using GeneRead FFPE Kit (Qiagen, Germantown, MD, USA) following the manufacturer’s instructions. Eluted DNA was assessed for purity using the Thermo Scientific NanoDrop™ 1000 Spectrophotometer, and double-stranded DNA was quantified using the Qubit Fluorometer (Thermo Fisher, Carlsbad, CA, USA).

### 4.7. Bisulfite Conversion and Quantitative Methylation Specific-PCR Analysis (qMSP)

Sodium bisulfite modification and purification of DNA extracted from cell lines and tumor samples were performed by using EpiTect Bisulfite kit (Qiagen) according to manufacturer’s instruction. Bisulfite-converted genomic DNA was amplified using qMSP. Primers/probe sets were designed to cover the unmethylated promoter region of the *ACTB* gene used as a reference gene as described previously [73] and are listed in Supplemental Table S2.

Ten-fold dilutions for four points (50–0.05 ng) of enzymatically methylated human genomic DNA (CpGenome^TM^ Universal Methylated DNA, Millipore, Bedford, MA, USA) were used to construct a calibration curve for *ACTB* (reference) and *KEAP1* (target) genes. qMSP reactions were performed in 384-well plates in triplicate and loaded on an ABI PRISM™ 7900HT Sequence Detection System (Thermo Fisher, Carlsbad, CA, USA) and SDS 2.4 software (Thermo Fisher, Applied Biosystems division) was used to analyze the runs. Relative methylation levels were calculated for each sample by dividing the average value from the triplicates between *KEAP1* and *ACTB*.

### 4.8. Mutation Analysis And Loss of Heterozygosity Analysis (LOH)

DNA obtained from the 47 carcinoid tumors of the lung was analyzed by fluorescence-based direct sequencing for the entire *KEAP1* gene coding region, including exon-intron boundaries and for the exon 2 of *NFE2L2* gene (Supplemental Table S3) as previously described [3]. Sequences were read with an ABI 3100 sequence detection system and analyzed using the Sequencing Analysis software v.3.7 (Thermo Fisher, Applied Biosystems). LOH analysis was performed using four microsatellite markers flanking the *KEAP1* [3] and products were genotyped using a ABI 3100 sequence detection system (Supplemental Table S4). Allele sizes were scored by ABI Genescan and Genotyper Software 3.7 (Thermo Fisher, Applied Biosystems division). LOH or allelic imbalance (AI) values were calculated using the following formula: (peak 1 height/peak2 height in tumor DNA)/(peak 1 height/peak 2 height in normal DNA) using a value of 0.5 to define LOH/AI [65].

### 4.9. Immunohistochemistry (IHC)

FFPE sections of 16 carcinoids (3 AC and 13 TC) were selected for IHC analysis using different antibodies (Supplemental Table S5). Detection was made by using EnVision TMFLEX+ detection kit (Dako, Glostrup, Denmark) following the manufacturer’s protocol and results were evaluated using the Olympus BX51 microscope. KEAP1 protein expression was evaluated for cytoplasm reactivity in tumor cells using a semi-quantitative system which combined staining intensity and the percentage of positive neoplastic cells. A final immunoreactivity staining score (IRS) was obtained by adding the score of percentage positivity and intensity. Samples were classified ad negative/weak staining (IRS <2) and moderated/strong staining (IRS ≥2).

### 4.10. Statistical Analysis

Patients baseline characteristics were reported as median and range or frequencies and percentages for continuous and categorical variables, respectively. Baseline comparisons between groups according to *KEAP1* methylation status were made using the Fisher or chi-square test for categorical variables, and the Mann-Whitney U-test for continuous variables.

Progression-free survival was defined as the time between surgery and the first progression, or death without progression, or date of last follow-up visit for patients alive without progression. We calculated median follow-up according to the reverse Kaplan-Meier technique [72]. Overall survival was defined as the time between surgery and death, or date of last follow-up visit for alive patients. Survival curves were estimated using the Kaplan–Meier method and significance was assessed with a log-rank test. Furthermore, univariate Cox proportional hazards regression models were estimated, both unadjusted and stratified by histological subtype. Risks were reported as hazards ratios (HR) along with their 95% confidence interval (CI).

Comparison of the mRNA expression level of NRF2-target enzymes between carcinoids cells lines and the expression levels of the proteins normalized to actin was performed using two-tailed Student’s t-test. Correlation between RT-qPCR and Western blot data was assessed by Pearson’s correlation coefficient. Experiments were repeated three times and representative experiments are shown. In the histograms, the values are presented as means ± standard error of the mean (SEM). Student’s t-test for unpaired data was used.

A *p*-value <0.05 was considered for statistical significance. All analyses were performed using SAS Release 9.1 (SAS Institute, Cary, NC, USA) and with SPSS for Windows, Version 24.0.

For densitometry analysis, the images were analyzed using ImageJ Software. For relative quantification, the optical density value was determined for same sized boxes drawn around antibody-stained bands. Normalization was performed using β-Actin antibody staining bands. The association between IHC and methylation was tested using *t*-test.

## 5. Conclusions

In conclusion, our findings of *KEAP1* methylation and related NRF2 pathway deregulation in lung carcinoids add important data to the poorly uncovered epi-profile of lung NET. Validations of these results are needed in larger studies to find a clear link with the patient’s follow-up and stratification.

## Figures and Tables

**Table 1:** Clinical-pathological features of lung carcinoid samples harboring *KEAP1* molecular lesions.

ID Sample	Histotypes	Gender	*KEAP1* (Meth and/or LOH ^1^)	Years at Diagnosis	UICC Stage	Progression (Y/N)	Status (D/A)	DFS (mos)	OS (mos)
LCCH01-322	AC	M	Meth	52	IIA	Y	A	84	120
LCCH01-328	AC	M	LOH	47	IIA	N	A	67	67
LCCH01-329	AC	M	LOH	49	IA	N	A	124	204
LCCH01-330	AC	F	Meth	67	IB	N	A	142	142
LCCH01-378/85	AC	M	Meth	71	IA	Y	D	12	23
UD-3	AC	M	Meth	44	IB	Y	D	60	132
UD-7	AC	M	Meth	61	IB	N	A	84	84
UD-18	AC	F	Meth	35	IB	Y	A	36	120
UD-20	AC	F	Meth	69	IIIA	N	A	108	108
UD-24	AC	F	Meth	69	IB	Y	D	12	28
LCCH01-315	TC	M	Meth, LOH	54	IA	N	A	203	288
LCCH01-377/35	TC	F	Meth, LOH	35	IB	N	A	52	52
LCCH01-313	TC	F	LOH	57	IA	N	A	36	36
LCCH01-327	TC	M	Meth, LOH	69	IA	N	A	66	66
LCCH01-320	TC	F	Meth	29	IB	N	A	157	157
LCCH01-323	TC	F	Meth, LOH	70	IA	N	A	132	132
LCCH01-325	TC	F	Meth	36	IA	N	A	124	124
LCCH01-327	TC	M	Meth	69	IA	N	A	66	66
LCCH01-321bis/380	TC	M	Meth	48	IB	N	A	172	172
LCCH01-333	TC	M	Meth	71	IA	N	A	108	108
LCCH01-311	TC	M	Meth	50	IA	N	A	91	91
LCCH01-54	TC	M	Meth, LOH	70	IA	N	A	47	47
UD-9	TC	M	Meth	64	IA	N	A	72	72
UD-10	TC	M	Meth	74	IA	Y	D	48	60
UD-11	TC	F	Meth	63	IA	N	A	60	60
UD-19	TC	F	Meth	65	IB	N	A	48	48
UD-25	TC	M	Meth	65	IA	N	A	24	24

AC, Atypical Lung Carcinoid; TC, Typical Lung Carcinoid. ^1^ LOH, (L) is defined if the allelic imbalance value (Q_LOH_) is ≤0.05. Meth, *KEAP1* promoter methylation; mos, months; F, Female; M, Male; D/A, Dead/Alive; OS, Overall Survival; DFS, Disease Free Survival.

**Table 2:** Clinical and pathological data of lung carcinoid patients cohort.

Characteristics	Category	All subjects (*N* = 47)
Age at diagnosis (years, median, IQR)	-	63, 19.75
Gender (*n*,%)	Female	26 (55.3%)
	Male	21 (44.7%)
Histotype subclassification (*n*,%)	Typical Carcinoid	30 (63.8%)
	Atypical Carcinoid	17 (36.2%)
T (*n*,%)	T1	31 (67%)
	T2	15 (33%)
N (*n*,%)	N0	41 (89.1%)
	N1	3 (6.5%)
	N2	2 (4.4%)
M (*n*,%)	M0	47 (100.00%)
Tumour stage (*n*,%)	IA - IB	42 (89.4%)
	IIA - IIB - IIIA	5 (10.6%)

Data are reported as frequencies and percentages. mos, months.

**Table 3:** Clinical pathological characteristics for patients with typical carcinoid (*n* = 30), according to. their KEAP1 methylation status.

Characteristics	Category	UM	M	*p*-Value *
Age at diagnosis (years, median (range))	-	63 (30–76)	64 (29–74)	0.755
Tumor_size (mm, median (range))	-	25 (10–40)	20 (10–45)	0.775
Gender	Female	11 (73%)	6 (40%)	0.07
	Male	4 (27%)	9 (60%)	
T	T1	10 (71%)	11 (73%)	1.000
	T2	4 (29%)	4 (27%)	
N	N0	13 (93%)	15 (100%)	0.483
	N1	1 (7%)	0 (0%)	
M	M0	15 (100%)	15 (100%)	n.a.
Tumor stage	IA - IB	14 (93%)	15 (100%)	1.000
	IIA-IIB-IIIA	1 (7%)	0 (0%)	

Data are reported as frequencies and percentages. * *p*-value from two-sample Mann Whitney test and Fisher exact test for continuous and categorical variables, respectively. UM = unmethylated; M = methylated. n.a. = not analyzed

**Table 4:** Clinical pathological characteristics for patients with atypical carcinoid (*n* = 17), according to their *KEAP1* methylation status.

Characteristics	Category	UM	M	*p*-Value *
Age at diagnosis (years, median (range))	---	53 (40–70)	64 (35–71)	0.736
Tumor size (mm, median (range))	---	20 (10–48)	32.5 (20–60)	0.132
Gender	Female	5 (56%)	4 (50%)	1.000
	Male	4 (44%)	4 (50%)	
T	T1	7 (78%)	3 (38%)	0.153
	T2	2 (22%)	5 (62%)	
N	N0	7 (78%)	6 (75%)	0.133
	N1	2 (22%)	0 (0%)	
	N2	0 (0%)	2 (25%)	
M	M0	9 (100%)	8 (100%)	n.a.
Tumor stage	IA-IB	7 (%)	6 (%)	0.893
	IIA-IIB-IIIA	2 (%)	2 (%)	

Data are reported as frequencies and percentages. * *p*-value from two-sample Mann Whitney test and Fisher exact test for continuous and categorical variables, respectively. UM = unmethylated; M = methylated. n.a. = not analyzed

**Table 5:** NRF2-interacting proteins identified in lung carcinoids.

Protein Name (symbol)	Reference
NAD(P)H dehydrogenase (quinone 1) (NQO1)	[21], this report
Aldo-Keto Reductase Family 1 (AKR1) (AKR1C1)	[22], this report
Hemoglobin subunit beta (HBB)	[23,24]
Ferritin light chain (FTL)	[24,25]
Ferritin heavy chain (FTH)	[24,26,27,28,29]
Superoxide dismutase (SOD)	[24,30,31,32,33,34,35]
Extracellular Superoxide dismutase (SODE)	[24,36,37]
Carbonic Anhydrase 1 (CAH1)	[24,38]
Annexin V (ANXA5)	[24,39,40]
Peroxiredoxin-2 (PRX2)	[24,41,42,43]
Peroxiredoxin-3 (PRX3)	[24,42,44,45]
Transthyretin (TTR)	[24,46]
Hemopexin (HPX)	[24,47]
Transaldolase (TALDO)	[24,48]
Heterogenous Nuclear Ribonucleo protein K (HNRPK)	[24,49,50]
Histone H4	[24,51,52,53,54]
Heat Shock Protein Hsp90-alpha (HSP90)	[24,55,56,57]
Elongation Factor 1-alpha1 (EF1A1)	[24,58]

## Supplementary Materials

Supplementary materials can be found at https://www.mdpi.com/1422-0067/20/10/2531/s1.
