# Supplementary material for: Effects of KEAP1 Silencing on the Regulation of NRF2 Activity in Neuroendocrine Lung Tumors
Source: Int J Mol Sci. 2019 May 23;20(10):2531. doi: 10.3390/ijms20102531 (PMC6566555; doi:10.3390/ijms20102531)

Table S1. Probes sets used for RT-qPCR analysis.

Gene name	Taqman gene expression assay Code
KEAP1	Hs00202227_m1*
AKR1C1	Hs04230636_sH*
NQO1	Hs02512143_s1*
TXN1	Hs01555214_g1*
RPLPO	4326314E*

*Taqman gene expression assay from Life Technologies, Thermo Fisher Inc.

Table S2. Primers/probes sets used for qMSP assays.

Primer/Probe name	Primer/Probe sequence (5 ‘ → 3’)	Annealing Temperature (℃)
KEAP1-meth_forw	TGCGGTCGTCGGATTACGAGGTCG	66
KEAP1-meth_rev	CTTCCATCTCCCGATTTCGTTAC	
KEAP1-meth_probe	FAM-GTGGCGCGTAGTTTCGCGAG-TAMRA	
ACTB-forw	TGGTGATGGAGGAGGTTTAGTAAGT	55
ACTB-rev	AACCAATAAAACCTACTCCTCCCTTAA	
ACTB-probe	FAM-ACCACCACCCAACACACAATAACAAACA[illegible] TAMRA	

Table S3. Primers sets used for *KEAP1* and *NFE2L2* genes mutation analysis.

Primer name	Primer sequence (5 ‘ → 3’)	Annealing Temperature (℃)
KEAP1-Ex-3aF<br>KEAP1-Ex-3aR	TTGCAAAACGAGGCCCCGGC<br>TGCACTCAGTGGAGGCGTAC	60
KEAP1-Ex-3bF<br>KEAP1-Ex-3bR	CTGCAGTCACAGTGCCCTGA<br>ACCTTGTGGGCCATGAACTG	60
KEAP1-Ex-3cF<br>KEAP1-Ex-3cR	GCCAGCAGCTGTGTGACGTC<br>ACTTCTCGCCCATGGAGATG	60
KEAP1-Ex-3dF<br>KEAP1-Ex-3dR	CCAAGGTCATGGAGCGCCTC<br>TGGTCCTTCTCCTGACACTG	60
KEAP1-Ex-4aF<br>KEAP1-Ex-4aR	GTGACTGGAGAGTCAGCCCG<br>ACTTCTGCAGCTGCATCTGC	60
KEAP1-Ex-4bF<br>KEAP1-Ex-4bR	CATCAACTGGGTCAAGTACG<br>GGTTGTAAGCCTCCAGGTAG	56
KEAP1-Ex-4cF<br>KEAP1-Ex-4cR	AGGTGGGCCGCCTGATCTAC<br>GCGACCACTGATTGGTCATG	56
KEAP1-Ex-4dF<br>KEAP1-Ex-4dR	AACTCGCCCGACGGCAACAC<br>GACTTGCCAGGAGCAGGACC	54
KEAP1-Ex-5F<br>KEAP1-Ex-5R	GTCAGCTATAATGGCCATTG<br>TGTTCCTGGGTGCTCCCCTC	60
KEAP1-Ex-6F<br>KEAP1-Ex-6R	TCCCAAAGCCAGACCCCCAG<br>AGATGGGCTAGTCAGGACTC	60
KEAP1-Ex-7aF<br>KEAP1-Ex-7aR	TCTTGGATGTGGTGTGACAG<br>TGATACTCCCCATTGGACTG	56
NFE2L2- Ex2-F<br>NFE2L2-Ex2-R	CCACCATCAACAGTGGCATA<br>CCTGCCATAACTTTCCCAAG	64

Table S4. Primers sets used for fluorescence-based loss of heterozygosity (LOH) analysis.

Primer name	Primer sequence (5 ‘ → 3’)	Annealing Temperature (℃)
D19S865_for	GCTATTTGGGGTCTCTATCAATG	60
D19S865_rev	GAAATCGCACAGTATTTGTCTCAC	
DM1_for	TGAAGCTGAGGCACAGG	60
DM1_rev	ATGATGGACACTACACCTTCA	
D19S906_for	AGATCGCACCACTGTACTCC	60
D19S906_rev	TTCCCGCCTAGTAACGGAC	
D19S840_for	ATAGGCCAAGACTGTCTAAAACAA	60
D19S840_rev	GCCCTAACTGCTGTAAGAGAACT	

Table S5. Antibodies used for western blot and immunohistochemical profiling of lung cancer cohort.

Antibody	Clone	Manufacturer	Source
KEAP1	Ag0779	Proteintech	Rabbit
NRF2	Ag9489	Proteintech	Rabbit
NQO1	Ag2009	Proteintech	Rabbit
TXNRD1	Ag1618	Proteintech	Rabbit

Supplemental Figure S1

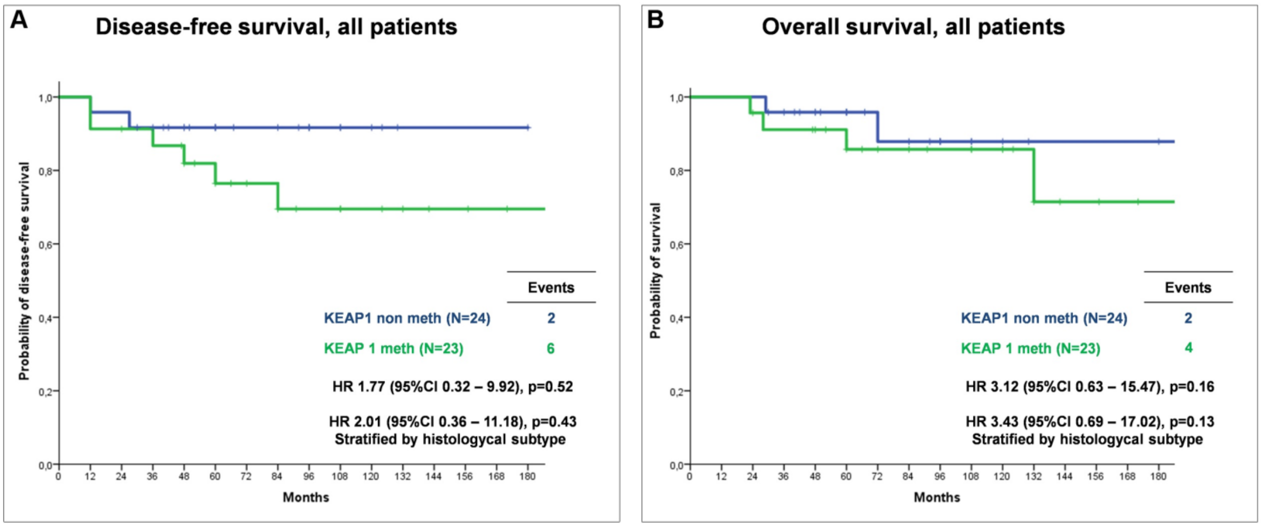

Supplement: Supplementary file 1 [file ijms-20-02531-s001.pdf]
